# Impact of COVID-related Discrimination on Psychological Distress and Sleep Disturbances across Race-Ethnicity

**DOI:** 10.1007/s40615-023-01614-5

**Published:** 2023-05-01

**Authors:** Paula D. Strassle, Miciah J. Wilkerson, Anita L. Stewart, Allana T. Forde, Chandra L. Jackson, Rupsha Singh, Anna María Nápoles

**Affiliations:** 1 Division of Intramural Research, National Institute on Minority Health and Health Disparities, National Institutes of Health, Bethesda, MD USA; 2 University of California San Francisco, Institute for Health & Aging, Center for Aging in Diverse Communities, San Francisco, CA USA; 3 Epidemiology Branch, National Institute of Environmental Health Sciences, National Institutes of Health, Research Triangle Park, NC USA

**Keywords:** COVID-19, Discrimination, Psychological distress, Sleep disturbances, Racial and ethnic minorities

## Abstract

COVID-related discrimination towards historically marginalized racial-ethnic groups in the United States has been well-documented; however, its impact on psychological distress and sleep (overall and within specific racial-ethnic groups) is largely unknown. We used data from our nationally representative, online survey of 5,500 American Indian/Alaska Native, Asian, Black/African American, Native Hawaiian/Pacific Islander, Latino, White, and multiracial adults, conducted from 12/2020-2/2021. Participants were asked how often they experienced discriminatory behaviors “because they think you might have COVID-19” (modified Everyday Discrimination Scale). Psychological distress was captured as having experienced anxiety-depression symptoms (Patient Health Questionairre-4, PHQ-4), perceived stress (modified Perceived Stress Scale), or loneliness-isolation (“How often have you felt lonely and isolated?”). Sleep disturbances were measured using the Patient-Reported Outcomes Information System Short Form Sleep Disturbance scale (PROMIS-SF 4a). Overall, 22.1% reported COVID-related discriminatory behaviors (sometimes/always: 9.7%; rarely: 12.4%). 48.4% of participants reported anxiety-depression symptoms (moderate/severe: 23.7% mild: 24.8%), 62.4% reported feeling stressed (moderate/severe: 34.3%; mild: 28,1%), 61.0% reported feeling lonely-isolated (fairly often/very often: 21.3%; almost never/sometimes: 39.7%), and 35.4% reported sleep disturbances (moderate/severe:19.8%; mild: 15.6%). Discrimination was only associated with increased psychological distress among racial-ethnic minorities. For example, COVID-related discrimination was strongly associated with anxiety-depression among Black/African American adults (mild: aOR=2.12, 95% CI=1.43-5.17; moderate/severe: aOR=5.19, 95% CI=3.35-8.05), but no association was observed among White or multiracial adults. Mitigating pandemic-related discrimination could help alleviate mental and sleep health disparities occurring among minoritized racial-ethnic groups.

**Supplementary Information:**

The online version contains supplementary material available at 10.1007/s40615-023-01614-5.

## Introduction

Since the beginning of the COVID-19 pandemic, there have been several reports on the high prevalence of COVID-related discrimination, particularly among Asian communities [1–3]. In 2020, there were over 2,800 reported incidents of anti-Asian hate crimes [4], representing the largest number of reported incidents since 2008 [5]. However, COVID-related discrimination has impacted almost all minoritized racial-ethnic groups in the United States. For example, findings from our COVID-19’s Unequal Racial Burden (CURB) survey, a nationally representative survey of adults living in the US, revealed almost 1 in 3 Asian adults, 1 in 4 American Indian/Alaska Native, Latino, 1 in 4 Native Hawaiian/Pacific Islander adults, and 1 in 5 Black/African American adults experienced COVID-related discrimination during the first year of the pandemic; moreover, among adults experiencing COVID-related discrimination, half reported that the experiences happened frequently [1].

Racial discrimination experienced by socially disadvantaged communities has been increasingly recognized as a contributor to mental and physical health disparities in the US [6]. Prior to the pandemic, discrimination was repeatedly associated with depressive symptoms, anxiety, and stress among historically minoritized racial-ethnic groups [6–11]. Racial discrimination prior to the pandemic was also associated with symptoms of poor sleep, including short sleep duration, difficulty initiating, and maintaining sleep [12, 13]. And while increases in the prevalence of psychological distress, perceived stress, and poor sleep have been observed during the pandemic, particularly among minoritized racial-ethnic communities [14–17], the role that COVID-related discrimination may play remains unclear. To date, the few studies which have assessed the potential impact of COVID-related discrimination on psychological distress and sleep have largely been restricted to Asian adults [2, 18, 19] or only included Asian, Black, Latino, and White adults [20]. To date, no study has assessed the impact of COVID-related discrimination on psychological distress or sleep problems among American Indian/Alaska Native, Native Hawaiian/Pacific Islander, or multiracial adults.

Given our findings of disparities in COVID-related discrimination, and the need to explore how COVID-related discrimination might impact health and well-being, the purpose of this study was to 1) estimate the overall association between COVID-related discrimination and psychological distress (anxiety-depression symptoms, perceived stress, and loneliness-isolation) and sleep disturbances using data from a nationally representative cohort of adults representing all major racial/ethnic groups in the U.S., and 2) assess whether the association between COVID-related discrimination, psychological distress, and sleep disturbances varied across racial/ethnic groups. We hypothesized that experiencing more frequent discrimination would be associated with greater psychological distress and poorer sleep, and that discrimination would impact racial/ethnic minorities more adversely, compared to White adults.

## Methods

### Study Population and Survey Development

The COVID-19’s Unequal Racial Burden (CURB) survey was administered by YouGov, a consumer research firm based in Palo Alto, CA, which uses a proprietary, opt-in survey panel comprised of over 1.8 million US residents to conduct nationally representative online surveys. Panel members are recruited through a variety of methods to ensure diversity, and then matched to a theoretical target sample for each survey. For this study, the target sample was drawn from the 2018 American Community Survey 1-year sample data, and included 1,000 Asian, 1,000 Black/African American, 1,000 Latino (including 500 Spanish-speaking), 1,000 White, 500 American Indian/Alaska Native, 500 Native Hawaiian/Pacific Islander, and 500 multiracial adults aged ≥18 years (*n*=5,500 total). Matched panel members who completed the online survey were then weighed to obtain a nationally representative sample within each racial/ethnic group (e.g., Asian participants were weighted to represent all Asians living in the United States). Details about the CURB survey development and sampling design have been published previously [1].

Surveys were completed online between December 8, 2020 and February 17, 2021. The National Institutes of Health Office of Institutional Review Board Operations determined that this study does not qualify as human subjects research because data were de-identified (IRB# 000166).

### COVID-Related Discrimination

COVID-related discrimination was measured using four questions; three were adapted from the Everyday Discrimination Scale [21] to be COVID-19-specific: 1) people acting afraid of you, 2) being called names or insulted, and 3) being threatened or harassed “because they think you might have COVID-19”. An additional question was created based on news reports that persons of Chinese descent were hearing racist comments from people thinking they were the cause of COVID-19. For all four items, participants were asked how often they experienced each type of discrimination using a 4-level response scale (1=never, 2=rarely, 3=sometimes, 4=always). Based on multitrait scaling analysis, *people acted afraid of you* was not highly correlated with the other three items (r=0.49) and was analyzed separately. The three other questions were combined into a *discriminatory behaviors* scale by averaging the responses. The *discriminatory behaviors* scale was then re-categorized as never (1), rarely (>1 to 2), sometimes (>2 to 3), and always (>3). Details on the *discriminatory behaviors* and *people acted afraid of you* measures developed from the CURB survey have been described previously [1]. For analyses, sometimes and always experiencing discrimination were collapsed into a single category for both measures due to relatively low prevalence of participants reporting “always” experiencing discrimination.

### Psychological Distress (Anxiety-Depression Symptoms, Perceived Stress, Loneliness-Isolation)

We used three measures to capture different aspects of psychological distress: anxiety-depression symptoms, perceived stress, and loneliness-isolation. All three of these measures have been described in more detail previously [14]. Anxiety-depression symptoms were measured using the validated 4-item Patient Health Questionnaire (PHQ-4) [22] which asks about being bothered by feeling nervous/anxious, not able to control worrying, little interest or pleasure, and feeling depressed or hopeless in the past two weeks. Summated scores (ranging from 0-12) were categorized as normal (0-2), mild (3-5), moderate (6-8), and severe (9-12) using the PHQ-4 scoring methodology. [22] Due to low prevalence, moderate and severe were combined into a single category for analyses.

Perceived stress was measured using a modified Perceived Stress Scale (PSS) [23]. The modified scale consisted of the six questions which asked how often participants felt stressed in the past month (e.g., unable to control important things; felt nervous and stressed; felt that could not cope). Response options for each item were never (1), almost never (2), sometimes (3), fairly often (4), and very often (5). Responses were averaged to create a perceived stress scale (range 1-5). The perceived stress scale was then categorized as low (1 to <2), mild (2 to <3), moderate (3 to <4), and severe stress (≥4). Due to low prevalence, moderate and severe stress were combined into a single category for analyses.

Loneliness was measured using a single question which asked, “In the past month, how often have you felt lonely and isolated?” Response options were collapsed and categorized as never (1), almost never/sometimes (2-3), and fairly often/very often (4-5).

### Sleep Disturbances

Sleep disturbances were assessed using the Patient-Reported Outcomes Information System Short Form Sleep Disturbance scale (PROMIS-SF v1.0 Sleep Disturbance 4a) [24], which asks participants about their sleep quality and frequency of sleep problems in the past seven days. Sleep disturbances was categorized as within normal limits (<55), mild (55.0-59.9), moderate (60.0-69.9), and severe (≥70) sleep disturbances using PROMIS 4a T-scores calculated using the non-missing responses to the four sleep questions. Again, moderate and severe sleep disturbances were combined into a single category for analyses due to relatively low prevalence. Supplemental Table 1 contains all the questions used to create the psychological distress and sleep disturbances measures.

### Other Covariates of Interest

Self-reported sociodemographics included race-ethnicity (American Indian/Alaska Native, Asian, Black/African American, Latino, Native Hawaiian/Pacific Islander, White, multiracial), age (18-34, 35-49, 50-64, ≥65 years old), gender (male, female, transgender or non-binary), English speaking proficiency (limited vs. not limited), highest education level (<high school graduate, high school graduate, >high school graduate), and family annual income (<$20K, $20-$59K, $60-99K, ≥$100K). Limited English proficiency was defined as being able to speak English “not at all,” “poorly,” or “fairly well” vs. “well” or “very well.” Missingness was minimal for all variables except for family annual income (*n*=659 [unweighted] selected “Prefer not to say”).

### Statistical Analyses

The prevalence of psychological distress (anxiety-depression symptoms, perceived stress, and loneliness-isolation) and sleep disturbances, stratified by frequency of COVID-related discrimination were estimated using descriptive statistics. Multinomial logistic regression was used to estimate the association between experiencing discrimination, psychological distress, and sleep disturbances. Models were adjusted for race/ethnicity, age group, gender, English proficiency, annual household income, and education. As a sensitivity analysis, we added anxiety-depression symptoms as a covariate when modeling perceived stress, loneliness-isolation, and sleep disturbances to account for potential negative affect bias.

To assess differences in the association between COVID-related discrimination, psychological distress, and sleep disturbances, separate models were run for each racial-ethnic group. Additionally, interaction terms between race-ethnicity and COVID-related discrimination were added to the models described above to obtain p-values assessing whether the association between COVID-related discrimination, psychological distress, and sleep disturbances differed by race-ethnicity. Because results from the two different modeling approaches (separate models for each race-ethnicity, interaction terms between COVID-related discrimination and race-ethnicity) produced nearly identical results, only the effect estimates from the race-ethnicity specific models are reported. For these models, COVID-related discrimination (*discriminatory behaviors* and *people acted afraid of you*) were dichotomized into any (rarely, sometimes, or always) vs. never.

All models were performed using SAS version 9.4 (SAS Institute, Inc., Cary, NC, USA). All analyses were weighted to produce nationally representative estimates within each racial/ethnic group and counts were rounded for interpretation.

## Results

The study included 5,804 online survey respondents (response rate: 20.0%) that were matched down to a sample of 5,500 to produce the final, weighted dataset. Demographics, overall and stratified by race/ethnicity, are reported in Table 1. Among all participants, 22.1% reported experiencing *discriminatory behaviors* (sometimes/always: 9.7%%; rarely: 12.4%) and 42.7% reported experiences of *people acted afraid of you* (sometimes/always: 22.6%; rarely: 20.1%), Supplemental Table 2. Substantial differences in the prevalence of COVID-related discrimination were seen across race/ethnicity, English proficiency, education level, household income, and geographic area; these results have been previously reported [1].

**Table 1:** Demographics and other participant characteristics, weighted to be nationally representative within each racial/ethnic group, among participants of the COVID-19’s Unequal Racial Burden (CURB) survey, December 2020 – February 2021, *n*=5,550

	Overall	American Indian/Alaska Native	Asian	Black/African American	Latino	Hawaiian/Pacific Islander	White	Multiracial
Total^a^, N	5,500	500	1000	1000	1000	500	1000	500
Age, years, median (IQR)	42 (29, 58)	43 (29, 60)	42 (30, 56)	41 (29, 57)	39 (28, 54)	40 (29, 54)	52 (34, 64)	35 (25, 52)
Gender, n (%)								
Male	2,588 (47.1)	231 (46.2)	456 (45.9)	472 (47.2)	490 (49.0)	236 (47.2)	475 (47.6)	228 (45.6)
Female	2,771 (50.5)	245 (49.1)	525 (52.8)	513 (51.3)	492 (49.2)	250 (50.0)	509 (50.9)	238 (47.5)
Non-binary^b^ or transgender	133 (2.4)	23 (4.6)	13 (1.3)	15 (1.5)	18 (1.8)	13 (2.6)	15 (1.5)	34 (6.8)
Health insurance, n (%)								
Any private	2,384 (43.6)	151 (31.3)	574 (58.8)	323 (33.5)	294 (31.1)	189 (39.4)	506 (53.0)	244 (50.3)
Public insurance only	1,953 (35.7)	227 (47.2)	253 (25.9)	422 (43.6)	282 (29.8)	204 (42.5)	327 (34.2)	167 (34.5)
Uninsured	1,137 (20.8)	103 (21.5)	148 (15.2)	221 (22.9)	371 (39.2)	88 (18.2)	122 (12.8)	73 (15.1)
Immigration status, n (%)								
US-born citizen	4,276 (77.8)	485 (97.2)	515 (51.5)	907 (90.7)	494 (49.4)	443 (88.7)	977 (97.8)	456 (91.1)
Foreign-born citizen\legal resident	946 (17.2)	14 (2.8)	433 (43.3)	83 (8.3)	301 (30.1)	50 (10.1)	21 (2.1)	43 (8.6)
Undocumented	275 (5.0)	0 (0.0)	52 (5.2)	10 (1.0)	204 (20.4)	6 (1.2)	1 (0.1)	1 (0.3)
Limited English proficiency^c^, n (%)	618 (11.2)	29 (5.9)	123 (12.3)	48 (4.8)	357 (35.7)	33 (6.6)	22 (2.2)	6 (1.1)
Education, n (%)								
Less than high school	498 (9.1)	57 (11.4)	40 (4.0)	68 (6.8)	188 (18.8)	41 (8.3)	55 (5.5)	49 (9.8)
High school/GED	1,791 (32.6)	192 (38.5)	229 (22.9)	375 (37.5)	387 (38.7)	196 (39.2)	291 (29.1)	120 (24.0)
Some college/vocational	1,690 (30.7)	181 (36.2)	215 (21.5)	351 (35.1)	274 (27.4)	178 (35.7)	312 (31.2)	180 (36.0)
College graduate or more	1,520 (27.6)	70 (13.9)	516 (51.6)	207 (20.7)	151 (15.1)	84 (16.8)	342 (34.2)	151 (30.1)
Family annual income^d^, n (%)								
<$20,000	1,095 (22.8)	123 (26.7)	106 (12.4)	292 (33.3)	247 (28.1)	110 (24.8)	130 (15.2)	86 (19.8)
$20,000-$59,999	1,921 (40.0)	193 (41.9)	281 (32.8)	346 (39.4)	423 (48.2)	165 (37.1)	336 (39.2)	177 (41.0)
$60,000-$99,999	974 (20.3)	81 (17.5)	229 (26.7)	137 (15.6)	139 (15.9)	108 (24.3)	181 (21.1)	100 (23.1)
≥$100,000	818 (17.0)	64 (13.9)	241 (28.1)	103 (11.8)	69 (7.9)	61 (13.7)	211 (24.6)	69 (16.0)
*Prefer not to say*	*692*	*39*	*144*	*122*	*122*	*56*	*142*	*68*
Married^e^, n (%)	2,551 (46.4)	238 (47.6)	507 (50.7)	318 (31.8)	533 (53.3)	248 (49.6)	522 (52.2)	186 (37.2)
Census division, n (%)								
New England	160 (2.9)	7 (1.5)	53 (5.3)	17 (1.7)	28 (2.8)	6 (1.2)	32 (3.2)	17 (3.4)
Middle Atlantic	622 (11.3)	14 (2.7)	163 (16.3)	131 (13.1)	100 (10.0)	8 (1.7)	152 (15.2)	54 (10.7)
East North Central	554 (10.1)	41 (8.1)	83 (8.3)	134 (13.4)	58 (5.8)	18 (3.6)	159 (15.9)	61 (12.2)
West North Central	224 (4.1)	35 (7.1)	23 (2.3)	31 (3.1)	18 (1.8)	8 (1.5)	94 (9.4)	16 (3.2)
South Atlantic	1,070 (19.5)	46 (9.2)	142 (14.2)	348 (34.8)	183 (18.3)	43 (8.6)	210 (21.0)	99 (19.8)
East South Central	252 (4.6)	19 (3.8)	10 (1.0)	117 (11.7)	14 (1.4)	7 (1.4)	63 (6.3)	22 (4.4)
West South Central	668 (12.2)	76 (15.3)	79 (7.9)	139 (13.9)	204 (20.4)	20 (4.1)	99 (9.9)	51 (10.2)
Mountain	555 (10.1)	113 (22.6)	71 (7.1)	29 (2.9)	97 (9.7)	91 (18.2)	93 (9.3)	60 (12.0)
Pacific	1,394 (25.4)	149 (29.8)	377 (37.7)	54 (5.4)	297 (29.7)	298 (59.7)	99 (9.9)	120 (24.0)
Residence urbanicity, n (%)								
Big city	1,433 (26.8)	99 (19.9)	265 (26.5)	344 (34.9)	376 (37.8)	98 (19.5)	130 (15.1)	122 (24.3)
Smaller city	1,022 (19.1)	92 (18.4)	161 (16.1)	193 (19.6)	266 (26.8)	89 (17.7)	134 (15.5)	87 (17.4)
Suburban area	1,664 (31.1)	101 (20.2)	415 (41.6)	300 (30.4)	228 (23.0)	153 (30.7)	285 (32.9)	182 (36.3)
Small town	659 (12.3)	94 (18.8)	106 (10.6)	83 (8.4)	67 (6.7)	98 (19.6)	150 (17.4)	62 (12.3)
Rural	566 (10.6)	114 (22.8)	53 (5.3)	67 (6.8)	56 (5.7)	62 (12.5)	166 (19.2)	48 (9.5)

Abbreviations: *IQR* interquartile range

^a^Online survey of US adults, weighted to be nationally representative within each racial/ethnic group; due to rounding percentages may not sum to 100%

^b^Includes individuals who identified as non-binary, gender queer, gender fluid, other, and none

^c^Limited English proficiency was defined as speaking English "not at all", "poorly", and "fairly well"

^d^Collected by YouGov at enrollment into panel and updated every 6 months

^e^Collected by YouGov at enrollment into panel and updated every 12 months

**Fig. 1:**
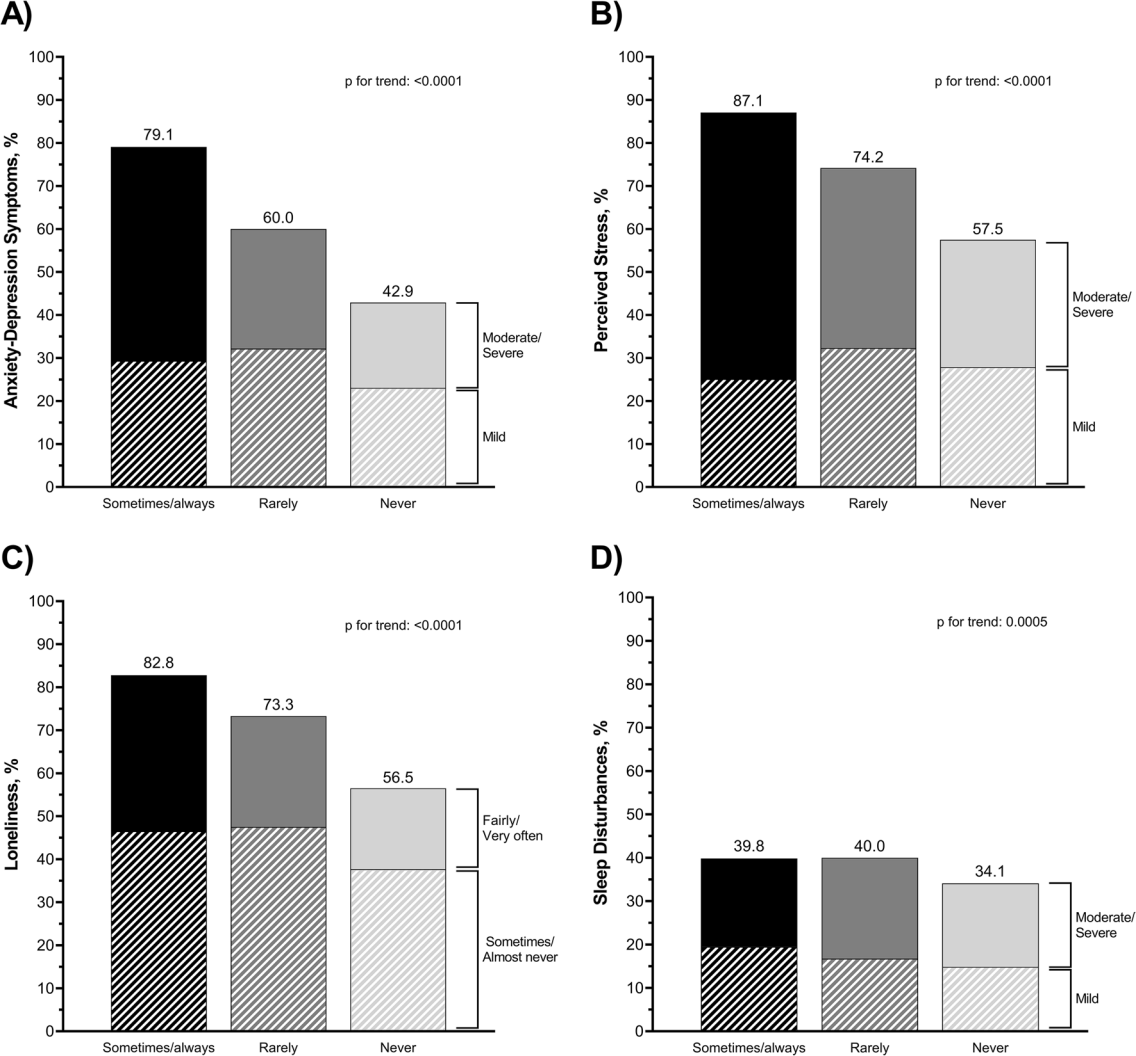
Prevalence of (**A**) anxiety-depression symptoms, (**B**) perceived stress, (**C**) loneliness-isolation, and (**D**) sleep disturbances, stratified by the frequency of experiencing discriminatory behaviors

Overall, 48.4% of participants reported anxiety-depression symptoms (moderate/severe: 23.7% mild: 24.8%), 62.4% reported feeling stressed (moderate/severe: 34.3%; mild: 28,1%), 61.0% reported feeling lonely (fairly often/very often: 21.3%; almost never/sometimes: 39.7%), and 35.4% reported sleep disturbances (moderate/severe:19.8%; mild: 15.6%), Supplemental Table 2. A clear dose-response trend was observed between *discriminatory behaviors* and psychological distress, p<0.0001 for all comparisons, Fig. 1A-C. Increased frequency of *discriminatory behaviors* was also associated with higher prevalence of sleep disturbances, *p*=0.0005, but the dose-response trend was less clear, Fig. 1D. Similar trends were seen between *people acted afraid of you*, psychological distress, and sleep disturbances, Supplemental Figure 1.

### Impact of COVID-Related Discrimination on Psychological Distress and Sleep Disturbances

After adjusting for sociodemographic characteristics, experiencing COVID-related discrimination was significantly associated with increased odds of anxiety-depression symptoms, perceived stress, and loneliness-isolation, with higher frequencies of discrimination being associated with worse outcomes, Table 2. For example, individuals who sometimes/always experienced *discriminatory behaviors* were three times as likely to have mild anxiety-depression symptoms (aOR=3.13, 95% CI=2.34-4.18) and seven times as likely to have moderate/severe anxiety-depression symptoms (aOR=7.00, 95% CI=5.31-9.23), compared to those who never experienced discrimination. Those who rarely experienced *discriminatory behaviors* were 70% more likely to report both mild and moderate/severe anxiety-depression symptoms (mild: aOR=1.69, 95% CI=1.36-2.11; moderate/severe: aOR=1.77, 95% CI=1.40-2.23), compared to never. Similar trends were seen between *discriminatory behaviors*, perceived stress, and loneliness-isolation. When anxiety-depression symptoms were included in the model, *discriminatory behaviors* was still significantly associated with increased odds of perceived stress and loneliness-isolation, although effect sizes were smaller, Supplemental Table 3. Experiencing *discriminatory behaviors* did not appear to increase the odds of sleep disturbances, after adjustment.

**Table 2:** Adjusted associations between experiencing COVID-related discrimination (discriminatory behaviors, people acted afraid of you) sometimes/always or rarely, compared to never, and anxiety-depressive symptoms, perceived stress, loneliness-isolation, and sleep disturbances, weighted to be nationally representative within racial-ethnic groups

	Discriminatory Behaviors^a^		People Acted Afraid of You	
	Sometimes/Always	Rarely	Sometimes/Always	Rarely
	aOR (95% CI)^b^	aOR (95% CI)^b^	aOR (95% CI)^b^	aOR (95% CI)^b^
Anxiety-depression symptoms^c^				
Moderate/severe	7.00 (5.31-9.23)	1.77 (1.40-2.23)	2.60 (2.16-3.14)	1.78 (1.47-2.17)
Mild	3.13 (2.34-4.18)	1.69 (1.36-2.11)	1.79 (1.48-2.16)	1.63 (1.35-1.96)
Perceived stress^d^				
Moderate/severe	7.97 (5.79-10.96)	2.14 (1.69-2.70)	4.05 (3.33-4.92)	2.02 (1.66-2.45)
Mild	2.92 (2.07-4.12)	1.77 (1.40-2.25)	2.25 (1.83-2.75)	1.88 (1.56-2.28)
Loneliness-isolation^e^				
Fairly often/very often	5.20 (3.81-7.11)	2.16 (1.67-2.80)	2.84 (2.31-3.50)	2.00 (1.61-2.48)
Almost never/sometimes	3.11 (2.34-4.13)	1.91 (1.53-2.38)	2.03 (1.70-2.43)	1.75 (1.47-2.09)
Sleep disturbances^f^				
Moderate/severe	1.15 (0.88-1.50)	1.35 (1.08-1.69)	1.50 (1.25-1.81)	1.35 (1.11-1.64)
Mild	1.29 (0.98-1.70)	1.09 (0.84-1.40)	1.02 (0.83-1.26)	1.24 (1.01-1.52)

Abbreviations: *aOR* adjusted odds ratio, *CI* confidence interval

^a^Discriminatory behaviors include being called names, being threatened/harassed, and hearing racist comments because people think you might have COVID-19

^b^Adjusted for race-ethnicity, age, gender, English proficiency, annual household income, and education

^c^Anxiety-depression was measured with the PHQ-4

^d^Perceived stress was assessed with a 6-item adapted version of the Perceived Stress Scale-10

^e^Loneliness was assessed with a single item that asks how often in the past month they felt lonely and isolated

^f^Sleep disturbances was assessed with the PROMIS-SF v1.0 Sleep Disturbance 4a

*People acted afraid of you* was also significantly associated with increased odds psychological distress, although the effect sizes were smaller compared to *discriminatory behaviors*, Table 2. Unlike *discriminatory behaviors*, *people acted afraid of you* was associated moderate/severe sleep disturbances (sometimes/always: aOR=1.50, 95% CI=1.25-1.81; rarely: aOR=1.35, 95% CI=1.11-1.64). After adjusting for anxiety-depression symptoms, the association between *people acted afraid of you* and *discriminatory behaviors* on perceived stress and loneliness-isolation were similar and *people acted afraid of you* was no longer associated with sleep disturbances, Supplemental Table 3.

### Race-Ethnicity Specific Impacts of COVID-Related Discrimination on Psychological Distress and Sleep Disturbances

**Fig. 2:**
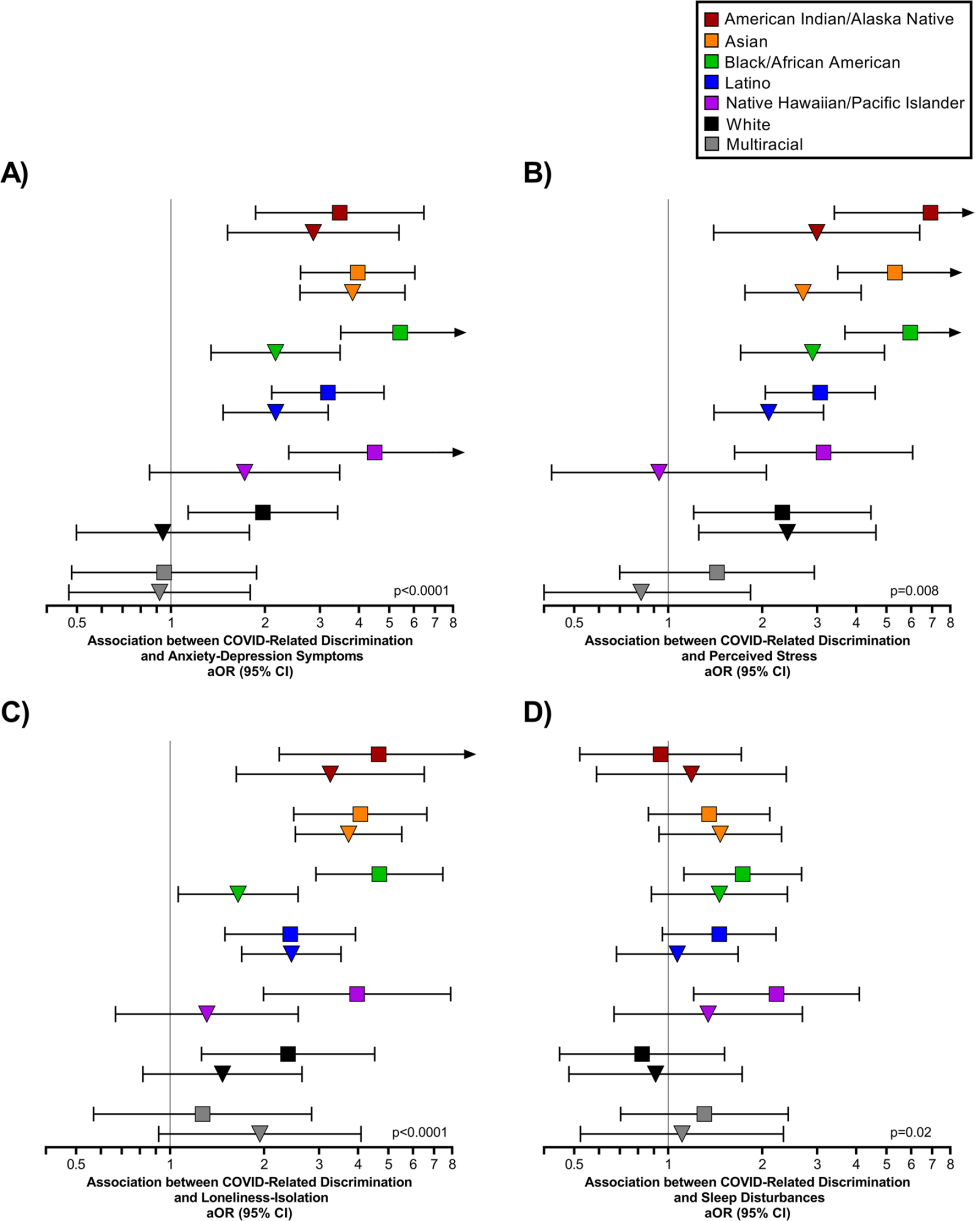
Race/ethnicity-stratified associations between experiencing any *discriminatory behaviors* and (**A**) anxiety-depression symptoms, (**B**) perceived stress, (**C**) loneliness-isolation and (**D**) sleep disturbances. The squares represent the association between *discriminatory behaviors* and moderate/severe (fairly often/very often) symptoms and the triangles represent the association between *discriminatory behaviors* and mild (almost never/sometimes) symptoms

When analyses were stratified by race-ethnicity, increased odds of psychological distress and sleep disturbances among individuals who experienced any *discriminatory behaviors* was largely only observed among historically minoritized racial-ethnic groups, Supplemental Table 4. For example, experiencing any COVID-related discrimination more than tripled the odds of moderate/severe anxiety-depression symptoms among American Indian/Alaska Native (aOR=3.47, 95% CI=1.87-6.46), Asian (aOR=3.96, 95% CI=2.60-6.04), Black/African American (aOR=5.42, 95% CI=3.50-8.39), Latino (aOR=3.18, 95% CI=2.10-4.81), and Native Hawaiian/Pacific Islander (aOR=4.49, 95% CI=2.38-8.46) adults, whereas a smaller association was observed among White adults (aOR=1.97, 95% CI=1.14-3.42), p<0.0001, Fig. 2A. Stronger associations between experiencing any *discriminatory behaviors*, perceived stress, and loneliness-isolation were also observed among racial-ethnic minorities, Fig. 2B and C.

COVID-related discrimination was only associated with increased odds of moderate/severe sleep disturbances among Black/African American (aOR=1.73, 95% CI=1.12-2.67) and Native Hawaiian/Pacific Islander (aOR=2.22, 95% CI=1.21-4.09) adults, Fig. 2D. COVID-related discrimination also appeared to increase the odds of moderate/severe sleep disturbances among Asian (aOR=1.35, 95% CI=0.86-2.11) and Latino (aOR=1.46, 95% CI=0.96-2.21) adults, but confidence intervals were wide.

Smaller differences and less clear trends were observed in the race-ethnicity stratified associations between *people acted afraid of you*, psychological distress, and sleep disturbances, Supplemental Table 5.

## Discussion

In a nationally representative cohort of adults representing all the major racial/ethnic groups in the United States, we found that increased COVID-related discrimination was associated with increased odds of psychological distress (anxiety-depression symptoms, perceived stress, loneliness-isolation) and sleep disturbances. Moreover, the more frequent the discrimination was experienced, the higher the likelihood the individual would have more severe psychological distress and disturbed sleep. When we stratified by race-ethnicity, COVID-related discrimination was only associated with psychological distress among American Indian/Alaska Native, Asian, Black/African American, Native Hawaiian/Pacific Islander, and Latino adults, and had minimal impact on White and multiracial adults. COVID-related discrimination was only associated with disturbed sleep among Black/African American and Native Hawaiian/Pacific Islander adults, and potentially Asian adults. Given that COVID-related discrimination was more likely to be experienced by racial-ethnic minorities [1] and COVID-related discrimination had a stronger association with psychological distress and sleep disturbances among racial-ethnic minority groups, discrimination could lead to worsening mental health and sleep disparities in the U.S. To date, this is the largest and most comprehensive assessment of the association between COVID-related discrimination, psychological distress, and sleep, and the first to include American Indian/Alaska Native, Native Hawaiian/Pacific Islander, and multiracial adults.

Currently, there is limited research on the impact of COVID-related discrimination in relation to psychological distress. To date, two studies have focused on Asian adults [18, 19], one focused on Chinese American adults [2], and one only included Asian, Black/African American, and Latino adults living in the South [20]. A few other studies have focused on specific communities, like international students [25], or looked more broadly at the impact of discrimination (COVID- and non-COVID-related) during the pandemic [26, 27]. Results have been consistent, with prior studies also finding that COVID-related discrimination negatively impacted mental health; however, our study highlights the disproportionately negative impact that COVID-related discrimination has on psychological distress (anxiety-depression symptoms, perceived stress, and loneliness-isolation) among minoritized racial-ethnic groups, including groups not previously studied, namely American Indian/Alaska Native, Native Hawaiian/Pacific Islander adults. Given this differential impact, COVID-related discrimination is likely to create or further exacerbate mental health disparities in the United States. Additionally, psychological distress has been associated with higher risk for chronic conditions like heart disease, cancer, and diabetes [28], suggesting that the long-term consequences of COVID-related discrimination on psychological distress, if unaddressed, could also worsen other health disparities.

Research on COVID-related discrimination and its impact on sleep health is also limited. To date, only one study has assessed the impact of COVID-related discrimination on sleep among Asian adults [19]. A few other analyses have examined discrimination (COVID- and non-COVID-related) during the pandemic and sleep among Asian, Black/African American, and White adults [29, 30]. All found that COVID-related discrimination was associated with sleep disturbances and shorter sleep time. COVID-related discrimination has also been shown to partially explain racial-ethnic disparities in sleep during the pandemic among 18–25-year-old adults [17]. We have added to this existing literature by not only including previously unstudied racial/ethnic populations (American Indian/Alaska Native, Latino, Native Hawaiian/Pacific Islander, and multiracial) but by also showing that the impact of COVID-related discrimination on sleep differs by race/ethnicity, primarily impacting Black/African American and Native Hawaiian/Pacific Islander adults. COVID-related discrimination may also worsen sleep among Asian and Latino adults, but our estimates were imprecise. Racial-ethnic minorities are also disproportionately affected by poor sleep quality, insufficient sleep, and sleep disorders [31], and COVID-related discrimination is likely to both exacerbate sleep health disparities as well as other downstream effects of poor sleep, including cardiovascular, metabolic, and mental health outcomes [32].

This study has some limitations. First, the survey was administered online, and individuals with limited internet access or familiarity with technology were probably less likely to participate. Although we did match and weight participants to obtain a nationally representative sample, it is possible that some selection bias existed. We also had a relatively low response rate for eligible panel members (20%). Additionally, in our prior analysis [1], we found that both lower income levels and lower educational levels were associated with higher rates of discrimination, which are also associated with reduced internet access and technology. Thus, it is possible that we may be underestimating the burden of COVID-related discrimination and the association between discrimination, mental health, and sleep, as some high-risk groups may have been less likely to participate. Second, the survey was administered only in English and Spanish (Latino participants only), and individuals who prefer a language other than English or Spanish were most likely unable or unwilling to participate. Third, sleep disturbances were self-reported, which is known to have some measurement error [33, 34]. Fourth, COVID-related discrimination was self-reported and based on each individual’s interpretation of the motivation behind the perpetrator’s behaviors. It is possible that some perceived discriminatory behaviors in fact had other motivations; however, we expect that even misattributed discrimination would have similar, negative impacts on mental health and sleep. Our modified Everyday Discrimination Scale may also not fully capture experiences of COVID-related discrimination during the pandemic. Psychological distress and sleep disturbances were also self-reported and may be subject to bias, although validated measures were used to capture anxiety-depression symptoms and perceived stress. Finally, this is a cross-sectional study and we were unable to control for all potential confounders, so some residual bias could still exist and results should not be interpreted as causal.

In summary, COVID-related discrimination towards minoritized racial-ethnic groups, particularly among Asian, American Indian/Alaska Native, Black/African American, Latino, and Native Hawaiian/Pacific Islander adults, has been widespread and prevalent. While prior studies have shown that COVID-related discrimination increases psychological distress and sleep disturbances, this is the first study to show that these associations are only experienced by racial/ethnic minorities and other marginalized communities, and not White adults. This study also represents one of the largest, most diverse, and nationally representative assessments on the impact of COVID-related discrimination on psychological distress and sleep, and provides valuable insights into the social and behavioral impacts of the COVID-19 pandemic, including the increased burden it has placed on racial-ethnic minorities in the U.S. Given these findings, it is likely that COVID-related discrimination will create or further exacerbate mental and sleep health disparities in the U.S., unless interventions and policies targeted at improved mental and physical health among at-risk communities are developed. For both mental health and sleep, culturally tailored resources should be developed, and policies and language surrounding future outbreaks and pandemics should be closely considered in order to minimize the risk of discrimination and its’ consequences on mental and physical health among communities most at risk. Moving forward, mitigating discrimination during outbreaks and pandemics should be of high importance, as it can have substantial mental and physical health consequences, as well as long-term detrimental effects on racial/ethnic minorities and other marginalized communities.

### Supplementary information

ESM 1 (DOCX 381 kb)
