# Supplementary material for: Impact of COVID-related Discrimination on Psychological Distress and Sleep Disturbances across Race-Ethnicity
Source: J Racial Ethn Health Disparities. 2023 May 1;11(3):1374–84. doi: 10.1007/s40615-023-01614-5 (PMC10150686; doi:10.1007/s40615-023-01614-5)
Supplement: Supplementary file 1 — (DOCX 381 kb) [file 40615_2023_1614_MOESM1_ESM.docx]

**Supplemental Table 1.** Survey questions and calculations of scale scores for psychological distress (anxiety-depression symptoms, perceived stress, loneliness-isolation) and sleep disturbances measures.

|  | **Survey Question(s)** | **Response Coding** | **Final measure** |
| --- | --- | --- | --- |
| **Anxiety-depression symptoms**  **(PHQ-4)^1,2^** | Over the last two weeks, how often have you been bothered by any of the following problems?   - Feeling nervous, anxious or on edge - Not being able to stop or control worrying - Little interest or pleasure in doing things - Feeling down, depressed, or hopeless | Nearly every day: 3  More than half the days: 2  Several days: 1  Not at all: 0 | Severe (9-12): 575 (10.5%)  Moderate (6-8): 727 (13.2%)  Mild (3-5): 1,361 (24.8%)  Normal (0-2): 2,826 (51.5%)  *Missing: 12* |
| **Perceived stress scale**  **(PSS)^3^** | In the past month, how often have you…   - Felt that you were unable to control important things in your life? - Felt nervous and “stressed”? - Found that you could not cope with all the things you had to do? - Been upset because of something that happened unexpectedly? - Been angered because of things that happened that were outside of your control? - Felt difficulties were piling up so high that you could not overcome them? | Very often: 5  Fairly often: 4  Sometimes: 3  Almost never: 2  Never: 1 | Low (1 to <2): 2,066 (37.6%)  Mild (2 to <3): 1,547 (28.1%)  Moderate (3 to <4): 1,286 (23.4%)  Severe: (≥4): 601 (10.9%)  *Missing: 0* |
| **Loneliness-Isolation** | In the past month, how often have you let lonely and isolated? | Very often: 5  Fairly often: 4  Sometimes: 3  Almost never: 2  Never: 1 | Very often: 624 (11.4%)  Fairly often: 548 (10.0%)  Sometimes: 1,272 (23.2%)  Almost never: 913 (16.6%)  Never: 2,135 (38.9%)  *Missing: 8* |
| **Sleep Disturbances**  **(PROMIS Short Form 4a)^4^** | In the past 7 days, my sleep quality was… | Very poor: 5  Poor: 4  Fair: 3  Good: 2  Very good: 1 | Within normal limits (<55): 3,554 (64.6%)  Mild (55.0-59.9): 855 (15.6%)  Moderate (60.0-69.9): 903 (16.4%)  Severe (≥70): 188 (3.4%)  *Missing: 0* |
|  | In the past 7 days…   - My sleep was refreshing (for example, felt rested)* - I had problems with my sleep - I had difficulty falling asleep | Very much: 5  Quite a bit: 4  Somewhat: 3  A little bit: 2  Not at all: 1 |  |

1. Kroenke K, Spitzer RL, Williams JB, Löwe B. An ultra-brief screening scale for anxiety and depression: the PHQ-4. *Psychosomatics*. 2009;50(6):613-21.
2. Löwe B, Wahl I, Rose M, et al. A 4-item measure of depression and anxiety: validation and standardization of the Patient Health Questionnaire-4 (PHQ-4) in the general population. *J Affect Disord*. Apr 2010;122(1-2):86-95.
3. Cohen S, Kamarck T, Mermelstein R. A global measure of perceived stress. *J Health Soc Behav*. Dec 1983;24(4):385-96.
4. Yu L, Buysse DJ, Germain A, et al. Development of short forms from the PROMIS™ sleep disturbance and Sleep-Related Impairment item banks. *Behav Sleep Med*. Dec 28 2011;10(1):6-24.

**Supplemental Table 2.** Prevalence of psychological distress (anxiety-depression symptoms, perceived stress, loneliness-isolation) and sleep disturbances, stratified by experiencing COVID-related discrimination (*discriminatory behaviors, people acted afraid of you*), among participants of the COVID-19’s Unequal Racial Burden (CURB) survey, conducted from December 2020 – February 2021, n=5,550.

|  |  | **Discriminatory Behaviors** | | |  | **People Acted Afraid of You** | | |
| --- | --- | --- | --- | --- | --- | --- | --- | --- |
|  | **Overall** | **Sometimes/**  **Always** | **Rarely** | **Never** |  | **Sometimes/**  **Always** | **Rarely** | **Never** |
| **Total, N (%)** | 5,500 | 534 (9.7) | 681 (12.4) | 4,286 (77.9) |  | 1,242 (22.6) | 1,104 (20.1) | 3,154 (57.4) |
| **Anxiety-depression symptoms** |  |  |  |  |  |  |  |  |
| Severe | 575 (10.5) | 93 (17.5) | 83 (12.2) | 400 (9.3) |  | 209 (16.9) | 93 (8.5) | 273 (8.7) |
| Moderate | 727 (13.2) | 171 (32.2) | 106 (15.6) | 450 (10.5) |  | 215 (17.4) | 204 (18.5) | 308 (9.8) |
| Mild | 1,361 (24.8) | 156 (29.4) | 218 (32.2) | 987 (23.1) |  | 338 (27.3) | 316 (28.7) | 706 (22.4) |
| Normal | 2,826 (51.5) | 111 (20.9) | 271 (40.0) | 2,443 (57.1) |  | 477 (38.5) | 487 (44.3) | 1,862 (59.1) |
| **Perceived stress** |  |  |  |  |  |  |  |  |
| Severe | 601 (10.9) | 99 (18.6) | 82 (12.0) | 420 (9.8) |  | 213 (17.2) | 106 (9.6) | 282 (8.9) |
| Moderate | 1,286 (23.4) | 232 (43.4) | 203 (29.9) | 851 (19.9) |  | 409 (32.9) | 302 (27.3) | 576 (18.3) |
| Mild | 1,547 (28.1) | 134 (25.1) | 220 (32.3) | 1,193 (27.8) |  | 341 (27.4) | 373 (33.8) | 833 (26.4) |
| Normal | 2,066 (37.6) | 69 (12.9) | 176 (25.8) | 1,821 (42.5) |  | 279 (22.5) | 324 (29.3) | 1,463 (46.4) |
| **Loneliness-isolation** |  |  |  |  |  |  |  |  |
| Very often | 624 (11.4) | 110 (20.7) | 88 (13.1) | 426 (9.9) |  | 199 (16.1) | 140 (12.7) | 284 (9.0) |
| Fairly often | 548 (10.0) | 82 (15.6) | 86 (12.7) | 380 (8.9) |  | 154 (12.5) | 129 (11.7) | 265 (8.4) |
| Sometimes | 1,272 (23.2) | 187 (35.4) | 179 (26.4) | 905 (21.1) |  | 366 (29.6) | 282 (25.6) | 623 (19.8) |
| Almost never | 913 (16.6) | 59 (11.1) | 143 (21.1) | 712 (16.6) |  | 180 (14.5) | 216 (19.6) | 517 (16.4) |
| Never | 2,135 (38.9) | 91 (17.2) | 181 (26.7) | 1,863 (43.5) |  | 337 (27.3) | 334 (30.3) | 1,463 (46.4) |
| **Sleep disturbances** |  |  |  |  |  |  |  |  |
| Severe | 188 (3.4) | 22 (4.2) | 30 (4.4) | 136 (3.2) |  | 64 (5.2) | 31 (2.8) | 93 (2.9) |
| Moderate | 903 (16.4) | 86 (16.2) | 129 (18.9) | 688 (16.1) |  | 238 (19.1) | 206 (18.7) | 459 (14.6) |
| Mild | 855 (15.6) | 104 (19.5) | 114 (16.7) | 637 (14.9) |  | 190 (15.3) | 199 (18.0) | 466 (14.8) |
| Within normal limits | 3,554 (64.6) | 321 (60.2) | 408 (60.0) | 2,825 (65.9) |  | 750 (60.4) | 668 (60.5) | 2,135 (67.7) |
| ^a^ | | | | | | | | |

**Supplemental Figure 1.** Prevalence of A) anxiety-depression symptoms, B) perceived stress, C) loneliness-isolation, and D) sleep disturbances, stratified by the frequency of *people acted afraid of you*.

**
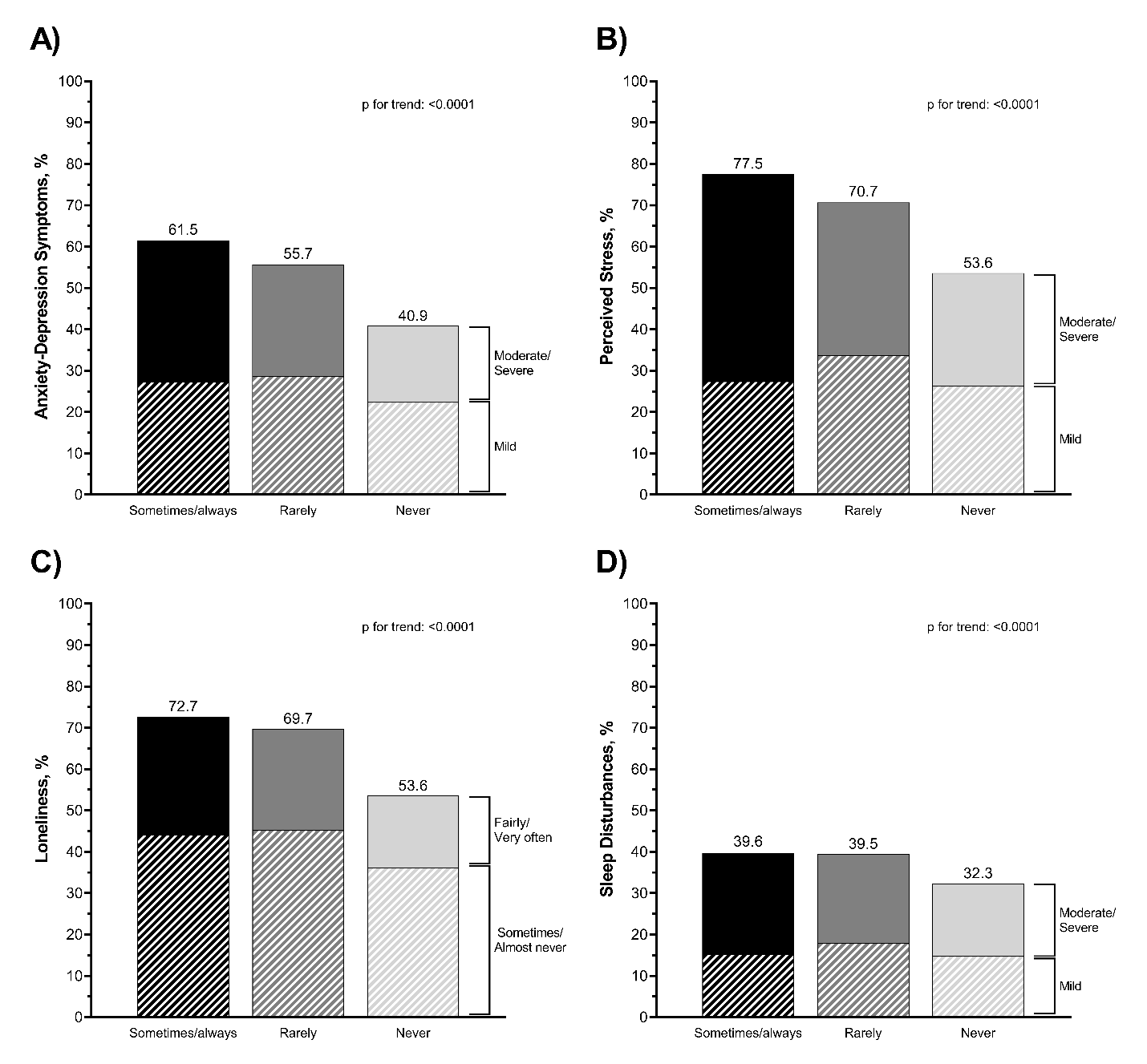
**

**Supplemental Table 3.** Adjusted associations between experiencing COVID-related discrimination (discriminatory behaviors, people acted afraid of you) sometimes/always or rarely, compared to never, and perceived stress, loneliness-isolation, and sleep disturbances, weighted to be nationally representative within racial-ethnic groups. Models were adjusted for anxiety-depression symptoms in order to account for potential negative affect bias.

|  | **Discriminatory Behaviors^a^** | |  | **People Acted Afraid of You** | |
| --- | --- | --- | --- | --- | --- |
|  | **Sometimes/Always** | **Rarely** |  | **Sometimes/Always** | **Rarely** |
|  | **aOR (95% CI)^b^** | **aOR (95% CI)^b^** |  | **aOR (95% CI)^b^** | **aOR (95% CI)^b^** |
| **Perceived stress^c^** |  |  |  |  |  |
| Moderate/severe | 3.54 (2.31, 5.42) | 1.79 (1.29, 2.47) |  | 3.92 (2.98, 5.14) | 1.64 (1.25, 2.15) |
| Mild | 1.97 (1.34, 2.89) | 1.57 (1.21, 2.05) |  | 2.25 (1.20, 2.82) | 1.69 (1.36, 2.09) |
| **Loneliness-isolation^d^** |  |  |  |  |  |
| Fairly often/very often | 1.65 (1.12, 2.43) | 1.82 (1.31, 2.52) |  | 1.81 (1.39, 2.37) | 1.60 (1.22, 2.11) |
| Almost never/sometimes | 1.69 (1.22, 2.33) | 1.68 (1.32, 2.14) |  | 1.66 (1.36, 2.03) | 1.54 (1.27, 1.88) |
| **Sleep disturbances^e^** |  |  |  |  |  |
| Moderate/severe | 0.49 (0.36, 0.66) | 1.09 (0.84, 1.40) |  | 0.98 (0.80, 1.21) | 1.08 (0.87, 1.34) |
| Mild | 0.76 (0.57, 1.02) | 0.93 (0.71, 1.21) |  | 0.80 (0.64, 1.00) | 1.06 (0.85, 1.32) |
| Abbreviations: aOR, adjusted odds ratio; CI, confidence interval  ^a^ Discriminatory behaviors include being called names, being threatened/harassed, and hearing racist comments because people think you might have COVID-19  ^b^ Adjusted for race-ethnicity, age, gender, English proficiency, annual household income, education, and anxiety-depression symptoms  ^c^ Perceived stress was assessed with a 6-item adapted version of the Perceived Stress Scale-10  ^d^ Loneliness was assessed with a single item that asks how often in the past month they felt lonely and isolated  ^e^ Sleep disturbances was assessed with the PROMIS-SF v1.0 Sleep Disturbance 4a | | | | | |

**Supplemental Table 4.** Race-ethnicity specific associations between experiencing any *discriminatory behaviors* on anxiety-depression symptoms, perceived stress, loneliness-isolation, and sleep disturbances. Separate models were run for each racial-ethnic group and interaction terms were used to calculate p-values.

|  | **American Indian/ Alaska Native** | **Asian** | **Black/African**  **American** | **Latino** | **Native Hawaiian/ Pacific Islander** | **White** | **Multiracial** |  |
| --- | --- | --- | --- | --- | --- | --- | --- | --- |
|  | **OR (95% CI)^a^** | **OR (95% CI)^a^** | **OR (95% CI)^a^** | **OR (95% CI)^a^** | **OR (95% CI)^a^** | **OR (95% CI)^a^** | **OR (95% CI)^a^** | **p-value^b^** |
| **Anxiety-depression symptoms** |  |  |  |  |  |  |  |  |
| Moderate/severe | 3.47 (1.87-6.46) | 3.96 (2.60-6.04) | 5.42 (3.50-8.39) | 3.18 (2.10-4.81) | 4.49 (2.38-8.46) | 1.97 (1.14-3.42) | 0.95 (0.48-1.88) | <0.0001 |
| Mild | 2.86 (1.52-5.38) | 3.81 (2.59-5.62) | 2.16 (1.34-3.48) | 2.16 (1.47-3.19) | 1.72 (0.85-3.47) | 0.94 (0.50-1.78) | 0.92 (0.47-1.70) |  |
| **Perceived stress** |  |  |  |  |  |  |  |  |
| Moderate/severe | 6.92 (3.41-14.06) | 5.32 (3.49-8.10) | 5.96 (3.68-9.64) | 3.07 (2.05-4.60) | 3.14 (1.63-6.06) | 2.32 (1.21-4.46) | 1.43 (0.70-2.94) | 0.008 |
| Mild | 2.99 (1.40-6.39) | 2.70 (1.76-4.15) | 2.90 (1.71-4.92) | 2.10 (1.40-3.14) | 0.93 (0.42-2.06) | 2.41 (1.25-4.63) | 0.82 (0.37-1.83) |  |
| **Loneliness-isolation** |  |  |  |  |  |  |  |  |
| Fairly often/very often | 4.66 (2.24-9.69) | 4.07 (2.49-6.64) | 4.68 (2.93-7.47) | 2.42 (1.50-3.92) | 3.97 (1.99-7.91) | 2.39 (1.26-4.52) | 1.27 (0.57-2.84) | <0.0001 |
| Almost never/sometimes | 3.26 (1.63-6.52) | 3.73 (2.52-5.52) | 1.65 (1.06-2.57) | 2.45 (1.70-3.52) | 1.31 (0.67-2.57) | 1.47 (0.82-2.64) | 1.94 (0.92-4.09) |  |
| **Sleep disturbances** |  |  |  |  |  |  |  |  |
| Moderate/severe | 0.95 (0.52-1.72) | 1.35 (0.86-2.11) | 1.73 (1.12-2.67) | 1.46 (0.96-2.21) | 2.22 (1.21-4.09) | 0.82 (0.45-1.52) | 1.31 (0.70-2.42) | 0.02 |
| Mild | 1.19 (0.59-2.39) | 1.47 (0.93-2.31) | 1.46 (0.88-2.41) | 1.07 (0.68-1.67) | 1.34 (0.67-2.69) | 0.91 (0.48-1.72) | 1.11 (0.52-2.34) |  |
| ^a^ Models were adjusted for age group, gender, English proficiency, annual household income, and education; discriminatory behaviors was modeled as any (rarely, sometimes, or always) vs. never; a separate model was run for each racial-ethnic group  ^b^ P-values were calculated by running a single model that included an interaction term between race-ethnicity and discriminatory behaviors | | | | | | | | |

**Supplemental Table 5.** Race-ethnicity specific associations between experiencing any *people acted afraid of you* on anxiety-depression symptoms, perceived stress, loneliness-isolation, and sleep disturbances. Separate models were run for each racial-ethnic group and interaction terms were used to calculate p-values.

|  | **American Indian/ Alaska Native** | **Asian** | **Black/African**  **American** | **Latino** | **Native Hawaiian/ Pacific Islander** | **White** | **Multiracial** |  |
| --- | --- | --- | --- | --- | --- | --- | --- | --- |
|  | **OR (95% CI)^a^** | **OR (95% CI)^a^** | **OR (95% CI)^a^** | **OR (95% CI)^a^** | **OR (95% CI)^a^** | **OR (95% CI)^a^** | **OR (95% CI)^a^** | **p-value^b^** |
| **Anxiety-depression symptoms** |  |  |  |  |  |  |  |  |
| Moderate/severe | 2.32 (1.41-3.82) | 2.33 (1.63-3.31) | 2.77 (1.91-4.01) | 1.95 (1.34-2.85) | 2.36 (1.41-3.94) | 2.28 (1.57-3.30) | 1.50 (0.91-2.49) | 0.47 |
| Mild | 1.73 (1.04-2.87) | 3.26 (2.19-4.85) | 2.40 (1.66-3.47) | 1.68 (1.34-2.85) | 1.83 (1.08-3.10) | 1.50 (1.03-2.18) | 1.38 (0.84-2.27) |  |
| **Perceived stress** |  |  |  |  |  |  |  |  |
| Moderate/severe | 4.29 (2.48-7.42) | 5.31 (3.63-7.78) | 3.68 (2.57-5.28) | 2.27 (1.60-3.23) | 2.18 (1.32-3.59) | 3.10 (2.08-4.63) | 2.15 (1.25-3.70) | 0.03 |
| Mild | 2.46 (1.39-4.36) | 2.99 (2.08-4.31) | 2.35 (1.60-3.45) | 1.66 (1.18-2.34) | 1.25 (0.74-2.11) | 3.06 (2.05-4.56) | 1.69 (0.95-2.99) |  |
| **Loneliness-isolation** |  |  |  |  |  |  |  |  |
| Fairly often/very often | 3.02 (1.75-5.24) | 3.21 (2.08-4.97) | 3.29 (2.22-4.92) | 2.16 (1.41-3.32) | 2.14 (1.23-3.72) | 2.67 (1.75-4.07) | 1.41 (0.81-2.46) | 0.19 |
| Almost never/sometimes | 2.62 (1.58-4.35) | 2.27 (1.65-3.13) | 1.95 (1.40-2.72) | 1.63 (1.20-2.22) | 1.62 (1.01-2.60) | 1.96 (1.37-2.80) | 1.62 (0.95-2.76) |  |
| **Sleep disturbances** |  |  |  |  |  |  |  |  |
| Moderate/severe | 1.55 (0.96-2.52) | 1.98 (1.30-3.01) | 1.79 (1.25-2.58) | 1.20 (0.82-1.76) | 2.05 (1.21-3.48) | 1.29 (0.88-1.89) | 1.33 (0.83-2.13) | 0.03 |
| Mild | 0.86 (0.48-1.54) | 1.10 (0.72-1.69) | 1.62 (1.06-2.47) | 1.05 (0.71-1.56) | 1.04 (0.61-1.77) | 1.34 (0.89-2.01) | 1.31 (0.75-2.29) |  |
| ^a^ Models were adjusted for age group, gender, English proficiency, annual household income, and education; people acted afraid of you was modeled as any (rarely, sometimes, or always) vs. never; a separate model was run for each racial-ethnic group  ^b^ P-values were calculated by running a single model that included an interaction term between race-ethnicity and people acted afraid of you | | | | | | | | |
